# Brazilian autoimmune encephalitis network (BrAIN): antibody profile and clinical characteristics from a multicenter study

**DOI:** 10.3389/fimmu.2023.1256480

**Published:** 2023-10-25

**Authors:** Bruna de Freitas Dias, Fabio Fieni Toso, Maria Eduarda Slhessarenko Fraife Barreto, René de Araújo Gleizer, Alessandra Dellavance, Pedro André Kowacs, Helio Teive, Mariana Spitz, Aline Freire Borges Juliano, Letícia Januzi de Almeida Rocha, Pedro Braga-Neto, Paulo Ribeiro Nóbrega, Jamary Oliveira-Filho, Ronaldo Maciel Dias, Clécio de Oliveira Godeiro Júnior, Fernanda Martins Maia, Rodrigo Barbosa Thomaz, Mara Lúcia Santos, Eduardo Sousa de Melo, Adaucto Wanderley da Nóbrega Júnior, Katia Lin, Orlando Graziani Povoas Barsottini, Verena Endmayr, Luís Eduardo Coelho Andrade, Romana Höftberger, Lívia Almeida Dutra

**Affiliations:** ^1^ Hospital Israelita Albert Einstein, São Paulo, Brazil; ^2^ Research and Development Division, Fleury Group, São Paulo, Brazil; ^3^ Instituto de Neurologia de Curitiba, Curitiba, Brazil; ^4^ Hospital Universitário da Universidade Federal do Paraná, Curitiba, Brazil; ^5^ Hospital Universitário Pedro Ernesto da Universidade Estadual do Rio de Janeiro, Rio de Janeiro, Brazil; ^6^ Hospital Universitário Presidente Dutra, São Luís do Maranhão, Brazil; ^7^ Hospital Universitário Professor Alberto Antunes, EBSERH, Universidade Federal do Alagoas, Maceió, Brazil; ^8^ Division of Neurology, Department of Clinical Medicine, Universidade Federal do Ceará, Fortaleza, Brazil; ^9^ Hospital Universitário Professor Edgard Santos, Salvador, Brazil; ^10^ Instituto Hospital de Base do Distrito Federal, Brasília, Brazil; ^11^ Hospital Universitário Onofre Lopes, Natal, Brazil; ^12^ Graduate Program in Medical Sciences, Universidade de Fortaleza, Fortaleza, Brazil; ^13^ Hospital Pequeno Príncipe, Curitiba, Brazil; ^14^ Hospital das Clínicas da Universidade Federal de Pernambuco, Recife, Brazil; ^15^ Universidade Federal de Santa Catarina, Florianópolis, Brazil; ^16^ Neurology and Ataxia Unit, Department of Neurology, Universidade Federal de Saão Paulo, São Paulo, Brazil; ^17^ Division of Neuropathology and Neurochemistry, Department of Neurology, Medical University of Vienna, Vienna, Austria; ^18^ Comprehensive Center for Clinical Neurosciences and Mental Health, Medical University of Vienna, Vienna, Austria; ^19^ Immunology Division, Fleury Group, São Paulo, Brazil

**Keywords:** autoimmune encephalitis, Anti-NMDA-receptor encephalitis, anti-glycine receptor antibody, encephalitis, antineuronal antibodies, anti-MOG antibodies

## Abstract

**Background:**

The frequency of antibodies in autoimmune encephalitis (AIE) may vary in different populations, however, data from developing countries are lacking. To describe the clinical profile of AIE in Brazil, and to evaluate seasonality and predictors of AIE in adult and pediatric patients.

**Methods:**

We evaluated patients with possible AIE from 17 centers of the Brazilian Autoimmune Encephalitis Network (BrAIN) between 2018 and 2022. CSF and serum were tested with TBAs and CBAs. Data on clinical presentation, complementary investigation, and treatment were compiled. Seasonality and predictors of AIE in adult and pediatric populations were analyzed.

**Results:**

Of the 564 patients, 145 (25.7%) were confirmed as seropositive, 69 (12.23%) were seronegative according to Graus, and 58% received immunotherapy. The median delay to diagnosis confirmation was 5.97 ± 10.3 months. No seasonality variation was observed after 55 months of enrolment. The following antibodies were found: anti-NMDAR (n=79, 54%), anti-MOG (n=14, 9%), anti-LGI1(n=12, 8%), anti-GAD (n=11, 7%), anti-GlyR (n=7, 4%), anti-Caspr2 (n=6, 4%), anti-AMPAR (n=4, 2%), anti-GABA-BR (n=4, 2%), anti-GABA-AR (n=2, 1%), anti-IgLON5 (n=1, 1%), and others (n=5, 3%). Predictors of seropositive AIE in the pediatric population (n=42) were decreased level of consciousness (p=0.04), and chorea (p=0.002). Among adults (n=103), predictors of seropositive AIE were movement disorders (p=0.0001), seizures (p=0.0001), autonomic instability (p=0.026), and memory impairment (p=0.001).

**Conclusion:**

Most common antibodies in Brazilian patients are anti-NMDAR, followed by anti-MOG and anti-LGI1. Only 26% of the possible AIE patients harbor antibodies, and 12% were seronegative AIE. Patients had a 6-month delay in diagnosis and no seasonality was found. Findings highlight the barriers to treating AIE in developing countries and indicate an opportunity for cost-effect analysis. In this scenario, some clinical manifestations help predict seropositive AIE such as decreased level of consciousness, chorea, and dystonia among children, and movement disorders and memory impairment among adults.

## Introduction

Autoimmune encephalitis (AIE) is a group of inflammatory diseases characterized by prominent neuropsychiatric symptoms associated with antineuronal and antiglial antibodies, usually directed against ion channel molecules or proteins associated with neurotransmitter receptors (1). The most common type of AIE is an anti-N-methyl-D-aspartate receptor (anti-NMDAR) encephalitis (2, 3). Patients with this condition are usually children or young adults, that present with subacute psychosis, memory complaints and/or movement disorders. A substantial proportion of patients become severely ill, presenting with a decreased level of consciousness, refractory status epilepticus, dysautonomia, or central hypoventilation (4). Diagnosis is based on clinical manifestations, complementary investigation, and antibody testing (3), according to the criteria of Graus. First-line treatment involves steroids, intravenous immunoglobulin (IVIg), and/or plasmapheresis (5–7), and variables associated with poor prognosis are delay in treatment over 4 weeks, CSF pleocytosis, abnormal brain MRI, and admission to an intensive care unit (8).

Reports from centers across different global regions show varying frequencies of AIE antibodies, such as anti-LGi1, anti-Caspr2, and anti-GABA-BR-associated encephalitis (9–11). For instance, Iran has a higher incidence of anti-GABA-BR-associated encephalitis when compared to other Asian countries and the United States (11). A study of the serum of 22,472 adult patients found that less than 4% of the samples harbor antineuronal antibodies, most commonly anti-NMDAR antibodies (24.6%), anti-GAD (21,5%) and anti-LGI1 antibodies (20,5%) (12). Among children (n=251), the most common antibodies detected were anti-NMDAR followed by anti-MOG (12). Interestingly, encephalitis associated with anti-MOG has been observed in both adult and pediatric populations.

AIE is not a rare cause of encephalitis (6). In developed countries, AIE prevalence was estimated in 8-13 cases/100.000 inhabitants, affecting people of all ages (13). Prior data from northern Europe showed that 20% of all encephalitis are immune-mediated (14) and in the California Encephalitis Project, 47% of encephalitis in patients under 30 years of age were identified as AIE (15). Moreover, reports from some centers or small geographic regions of non-tropical countries suggested that AIE might be seasonal, with higher frequency in warmer months (16, 17).

Information on AIE from developing countries is scarce. A review of all reported cases of AIE in Latin America published in 2020 included only 383 patients, probably due to underdiagnosis (18). Barriers to AIE diagnosis include limited access to testing. Over the past four years, we have established the Brazilian Autoimmune Encephalitis Network, which involves 17 sites in partnership with the Medical University of Vienna. We aimed to describe the clinical manifestations of seropositive Brazilian patients, evaluate AIE seasonality from a tropical country, and identify clinical variables as predictors of AIE in adult and pediatric patients.

## Material and methods

Brazilian Autoimmune Encephalitis Network (BrAIN) comprises 17 sites from all geographic Brazilian regions (including the states of Ceará, Alagoas, Pernambuco, Bahia, Maranhão, Rio Grande do Norte, Distrito Federal, Rio de Janeiro, São Paulo, Paraná, and Santa Catarina). Principal investigators from each Neurology Department’s site included patients from June 2018 to November 2022 with the following inclusion criteria: (1) possible AIE according to Graus (3) and (2) agreement to informed consent. This study was approved by the local Ethical Committee and was supported by the Medical University of Vienna, Fleury Group, and FAPESP.

Clinical and epidemiological information was compiled using REDCap. Data on gender, age at onset, clinical presentation, behavioral, cognitive, and motor symptoms, and initial treatment were collected. Additionally, data from complementary investigations such as CSF analysis, EEG, and brain MRI were recorded. The coordinator center further reviewed all information for a final decision on eligibility for antibody testing. Upon decision from the coordinating team, CSF and serum samples were frozen to -20°C and shipped to the coordinating center in São Paulo and then sent to the Division of Neuropathology and Neurochemistry, Department of Neurology, Medical University of Vienna.

In the laboratory in Vienna, serum and CSF samples of all patients included were screened for antineuronal autoantibodies (ANeA) using in-house tissue-based assays (TBA) for surface and intracellular antibodies. Samples showing a specific tissue staining in the TBA were sequentially tested using in-house or commercial cell-based assays (CBA) for surface antibodies (anti-NMDAR, anti-AMPAR, anti-GABA-BR, anti-GABA-AR, anti-LGI1, anti-Caspr2, anti-DPPX, anti- IgLON5, anti-neurexin3alpha, anti-mGluR1, and anti-mGluR5) and immunoblot (Ravo PNS 14 Line Assay), according to previously described protocols (19). Under the disclosure of the coordinator center, anti-MOG and anti-GlyR CBA were performed as previously described using in-house CBAs (20, 21). Anti-GAD was detected using TBA and immunoblot (20). Anti-MOG antibodies were screened in serum and if available CSF with an in-house CBA using full-length human MOG. Serum samples were scored according to a high-titer cut-off as MOG reactivity below cut-off (titer 1:40-1:80), moderately positive (titer 1:160-1:320), and strongly positive (titer 1:640 and higher).

Patients under 13 years of age were allocated to the pediatric cohort. After receiving the antibody results, the coordinator site classified adult and pediatric participants into 3 groups: seropositive (SP) AIE, seronegative (SN) AIE, and non-AIE. SP and SN AIE were diagnosed according to Graus criteria (3, 21). Because we wanted to evaluate SN patients according to Graus criteria, patients fulfilling the criteria of seronegative limbic encephalitis were not included in the SN group. All charts were reviewed for clinical phenotype characterization. We only included in the SN group patients fulfilling criteria for probable negative AIE criteria according to Graus.

**Figure 1:**
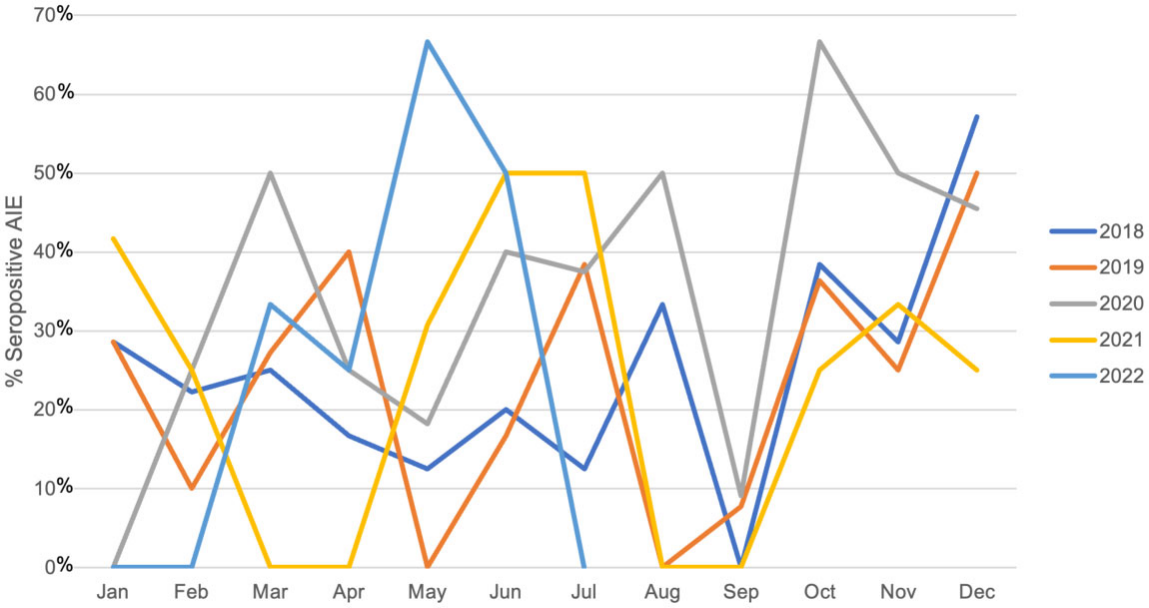
Seasonality of seropositive patients during 2018 and 2022 months using time series analysis of Ljung-Box.

Clinical, epidemiological, laboratory, and imaging characteristics were compared according to seropositivity using Mann-Whitney, chi-square, or Fisher’s exact test. AIE seasonality was based on the date of symptoms onset and was evaluated over the 55 months of the study, using the time series analysis of Ljung-Box **Figure 1**. Variables associated with SP AIE in adult and pediatric cohorts were evaluated with univariate analysis followed by logistic regression models. We used SPSS, R, and seatest for the statistical analysis. P-value < 0.05 was considered significant.

## Results

### The general profile of Brazilian seropositive patients

We tested 564 patients with possible AIE, 145 (26%) were confirmed as SP AIE, 69 (12%) were classified as SN AIE, 16 (3%) were classified as seronegative limbic encephalitis, and 334 (59%) had another diagnosis. Twenty-seven SP patients presented limbic encephalitis, including two anti-GABA-BR, one anti-GABA-AR, four anti-GAD, two anti-Caspr2, and four anti-LGI1. All SN patients presented with antibody-negative but probable AIE (ANPRA) phenotype.

Forty-two (29%) SP patients were children. The median delay to diagnosis confirmation was 5.97 ± 10.3 months. Sixty-eight patients were from southeast Brazil, 48 from the Northeast region, 14 from the South, 12 from the Midwest, and 3 from the North region. No seasonal variation for SP AIE diagnosis was observed within 55 months of patient enrolment (p= 0.245) (**Figure 1**).

Antibodies found in this cohort were 79 (54%) anti-NMDAR, 14 (10%) anti-MOG, 12 (8%) anti-LGI1, 11 (8%) anti-GAD, 7 (5%) anti-GlyR, 6 (4%) anti-Caspr2, 4 (3%) anti-AMPAR, 4 (3%) anti-GABA-BR, 2 (1%) anti-GABA-AR, 1 (1%) anti-IgLON5, and 5 others (1 anti-mGluR1, 1 atypical neuropil staining, 1 anti-Neurexin-3-alpha, 1 GFAP, and 1 anti-mGluR5) (**Supplemental Figure**).

Among pediatric SP patients, 55% were female with a mean age of 5.87 ± 2.9 years. Thirty children (71%) had anti-NMDAR, 11 (26%) had anti-MOG, and 1 (2%) had anti-GlyR. Of the 103 SP adult patients, 64% were female, with a mean age of 34.33 ± 20.33 years. Among adults, we found 49 anti-NMDAR patients (47%), 12 anti-LGI1 (11%), 11 anti-GAD (11%), 6 anti-GlyR (6%), 5 anti-Caspr2 (5%), and all the other antibodies described in this cohort.

Five patients presented a co-occurrence of ANeA. Two had anti-NMDAR and anti-MOG, 1 had anti-LGI1 and anti-Caspr2 antibodies, 1 had anti-GFAP and anti-AMPA, and 1 had anti-GlyR and anti-MOG. Ninety-nine patients (68%) had ANeA in both CSF and serum and 20 (14%) had only in serum (1 anti-GABA-BR, 11 anti-MOG, 1 anti-LGI1, and 7 anti-GlyR). Additionally, we found 9 patients with ANeA exclusively in the CSF (8 anti-NMDAR, and 1 anti-MOG).

We found four patients with possible AIE with high-risk antibodies (two with anti-Hu antibodies and two with anti-Yo). We excluded them from this analysis because of their distinct pathophysiology.

### Clinical characterization and complementary investigation

**Figure 2:**
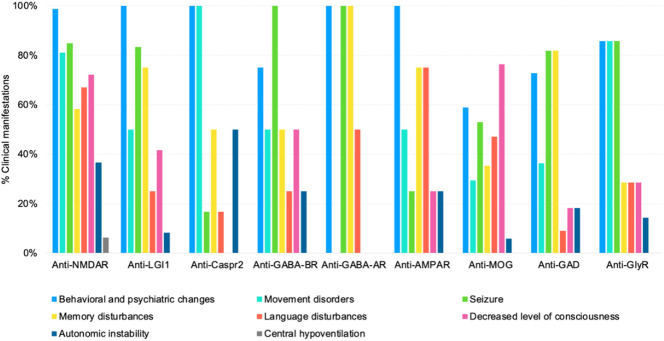
Clinical manifestations by the antineuronal antibody. NMDAR, N-methyl-D-Aspartate receptor; LGI1, Leucine-rich glioma inactivated 1; CASPR2, Contactin-associated protein 2; GABA-BR, Gamma-aminobutyric acid-B receptor; GABA-AR, Gamma-aminobutyric acid-A receptor; AMPAR, a-amino-3-hydroxy-5-methyl-4-isoxazole propionic acid receptor; MOG, Myelin oligodendrocyte glycoprotein; GAD, Glutamic acid decarboxylase; GlyR, Glycine receptor.

Among anti-NMDAR patients, 67% (n=53) were female and 38% (n=30) were children. All patients had the typical clinical picture, with behavioral symptoms, seizures, and/or movement disorders mostly orofacial dyskinesia (n=34), and dystonia (n=31). EEG showed diffuse slowing or disruption of base activity in 42 patients. All patients had anti-NMDAR antibodies in CSF. Brain MRI was normal in 57% of patients and 14% of patients had uni or bilateral mesial temporal FLAIR/T2 abnormalities. **Figure 2**, illustrates the distribution of symptoms by antibody and **Table 1** provides an overview of all clinical presentations and complementary investigation.

**Table 1:** Clinical and complementary investigation findings by each AIE phenotype.

Antibody (n)	Anti-NMDAR (79)	Anti-LGI1 (12)	Anti-Caspr2 (6)	Anti- GABA-BR (4)	Anti- GABA-AR (2)	Anti-AMPAR (4)	Anti-MOG (14)	Anti-IgLON5 (1)	Anti-GAD (11)	Anti-GlyR (7)
*Age (mean ± SD)*	26.17 ± 21.52	58.56 ± 16.73	39.54 ± 16.54	60.3 ± 16.64	59.29 ± 14.89	24.56 ± 8.75	15.78 ± 21.46	62	41.04 ± 15.36	23.25 ± 13.73
Complementary investigation n (%)										
Normal EEG	10 (13)	3 (25)	4 (67)	0 (0)	0 (0)	0 (0)	3 (21)	0 (0)	2 (18)	0 (0)
Normal Brain MRI	45 (57)	3 (25)	3 (50)	2 (50)	0 (0)	2 (50)	3 (21)	0 (0)	5 (45)	3 (43)
MTH	12 (15)	7 (58)	2 (33)	2 (50)	1 (50)	0 (0)	0 (0)	0 (0)	5 (45)	1 (14)
CH	14 (18)	0 (0)	0 (0)	0 (0)	1 (50)	1 (25)	8 (57)	0 (0)	0 (0)	2 (28)
WMH	9 (11)	0 (0)	0 (0)	0 (0)	0 (0)	0 (0)	5 (36)	0 (0)	1 (9)	1 (14)
Encephalic atrophy	5 (6)	0 (0)	0 (0)	0 (0)	0 (0)	2 (50)	0 (0)	0 (0)	1 (9)	0 (0)
*CSF pleocytosis n (%)*	48 (60)	2 (17)	2 (33)	1 (25)	0 (0)	1 (25)	7 (50)	1 (100)	1 (1)	4 (57)

NMDAR, N-methyl-D-Aspartate receptor; LGI1, Leucine-rich glioma inactivated 1; CASPR2, Contactin-associated protein 2; GABA-BR, Gamma-aminobutyric acid-B receptor; GABA-AR, Gamma-aminobutyric acid-A receptor; AMPAR, a-amino-3 hydroxy-5-methyl-4-isoxazole propionic acid receptor; MOG, Myelin oligodendrocyte glycoprotein; IgLON5, Immunoglobulin-like cell adhesion molecule 5; GAD, Glutamic acid decarboxylase; GlyR, Glycine receptor; BPC, Behavioral and psychiatric changes; DLC, Decreased level of consciousness; MTH, Mesial temporal hyperintensities; CH, Cortical hyperintensities; WMH, White matter hyperintensities.

Anti-LGI1 patients mostly presented behavioral and psychiatric changes, seizures, and memory disturbances. Most patients with anti-Caspr2 were male and one was female. All of them presented behavioral and psychiatric symptoms, as well as abnormal movements such as dyskinesia, dystonia, or myoclonus, and half of them showed autonomic instability.

All anti-GABA-BR patients presented seizures as the main clinical manifestation and half of them fulfilled the criteria for limbic encephalitis with bilateral mesial temporal hyperintensities in brain MRI. Anti-GABA-AR patients presented behavioral changes, seizures, and brain MRI with mesial temporal or cortical hyperintensities. Half of them had limbic encephalitis and 50% had normal MRI. Anti-AMPAR encephalitis patients presented behavioral complaints, memory, and language disturbance. Half of our patients presented with normal brain MRI, which is an interesting finding.

Of the 14 patients with anti-MOG, 11 were children and 3 were adults. Of note, anti-MOG represented 3% of all SP AIE adult patients and 26% of the children with AIE. Common manifestations were decreased level of consciousness, behavioral and psychiatric changes, and seizures. All patients had serum anti-MOG antibodies at a title higher than 1:80 (in-house CBA). One adult patient met both acute disseminated encephalomyelitis (ADEM) and AIE criteria, and the other two only met the criteria for AIE. Three patients (two adults and one child) had normal brain MRIs, including an 80-year-old female, who presented with memory complaints, hallucinations, and weight loss with a serum antibody titer of 1:160. Additionally, two adult patients were retrospectively diagnosed with anti-MOG encephalitis, with prior negative ANeA testing.

Concerning anti-GAD patients (n=11), four patients presented bilateral mesial temporal hyperintensities, compatible with limbic encephalitis, five had normal brain MRI, one patient with white matter hyperintensities, and another with unilateral mesial temporal hyperintensities.

Anti-GlyR patients (n=7) were mostly adults, and only one was a six-year-old child, who presented acute epilepsy and psychosis, that evolved with abnormal inferior limb posture and ataxia. Only three were male. All patients met AIE criteria and had only anti-GlyR antibodies detected. All developed variable abnormal movements such as ataxia, dyskinesia, tremor, and myokymia. One adult patient had a prior diagnosis of temporal lobe epilepsy with hippocampal atrophy and developed cephalic tremor, ataxia, and dystonia after two years. All of them had epilepsy. EEG findings were abnormal in all cases, mostly focal or diffuse slowing.

### Evaluation of prodromal symptoms amongst patients with AIE

**Table 2** shows the frequency of prodromal symptoms associated with each antibody. Anti-NMDAR, anti-MOG, and anti-GlyR patients showed the highest frequency of prodromal symptoms.

**Table 2:** Frequency of prodromal symptoms by antibody.

Antibody (n) (%)	Anti-NMDAR (79)	Anti-LGI1 (12)	Anti-Caspr2 (6)	Anti-GABA-BR (4)	Anti-GABA-AR (2)	Anti-AMPAR (4)	Anti-MOG (14)	Anti-IgLON5 (1)	Anti-GAD (11)	Anti-GlyR (7)
Fever *n* (%)	18 (23)	0 (0)	0 (0)	1 (25)	0 (0)	1 (25)	6 (35)	0/0	0 (0)	2 (28)
Nausea *n* (%)	10 (13)	0 (0)	0 (0)	0 (0)	0 (0)	1 (25)	3 (21)	0/0	1 (9)	1 (14)
Coryza *n* (%)	5 (6)	0 (0)	0 (0)	0 (0)	0 (0)	1 (25)	1 (7)	0/0	0 (0)	0 (0)
Cough *n* (%)	3 (4)	0 (0)	0 (0)	1 (25)	0 (0)	1 (25)	1 (7)	0/0	0 (0)	1 (14)
Headache *n* (%)	18 (23)	1 (8)	1 (17)	0 (0)	0 (0)	1 (25)	3 (21)	0/0	0 (0)	2 (28)
Malaise *n* (%)	7 (9)	0 (0)	0 (0)	0 (0)	0 (0)	0 (0)	1 (7)	0/0	1 (9)	0 (0)
Myalgia *n* (%)	1 (1)	1 (8)	0 (0)	0 (0)	0 (0)	0 (0)	1 (7)	0/0	0 (0)	1 (14)

NMDAR, N-methyl-D-Aspartate receptor; LGI1, Leucine-rich glioma inactivated 1; CASPR2, Contactin-associated protein 2; GABA-BR, Gamma-aminobutyric acid-B receptor; GABA-AR, Gamma-aminobutyric acid-A receptor; AMPAR, a-amino-3-hydroxy-5-methyl-4-isoxazole propionic acid receptor; MOG, Myelin oligodendrocyte glycoprotein; IgLON5, Immunoglobulin-like cell adhesion molecule 5; GAD, Glutamic acid decarboxylase; GlyR, Glycine receptor.

### Tumor screening and treatment data

Of the 529 possible AIE patients, 384 (72%) received immunotherapy by the time of enrollment. Intravenous methylprednisolone was the most prescribed treatment (n=339; 64%), followed by IVIg (n=208; 39%), plasmapheresis (n=31; 6%), rituximab (n=21; 4%), and cyclophosphamide (n=21; 4%).

**Figure 3:**
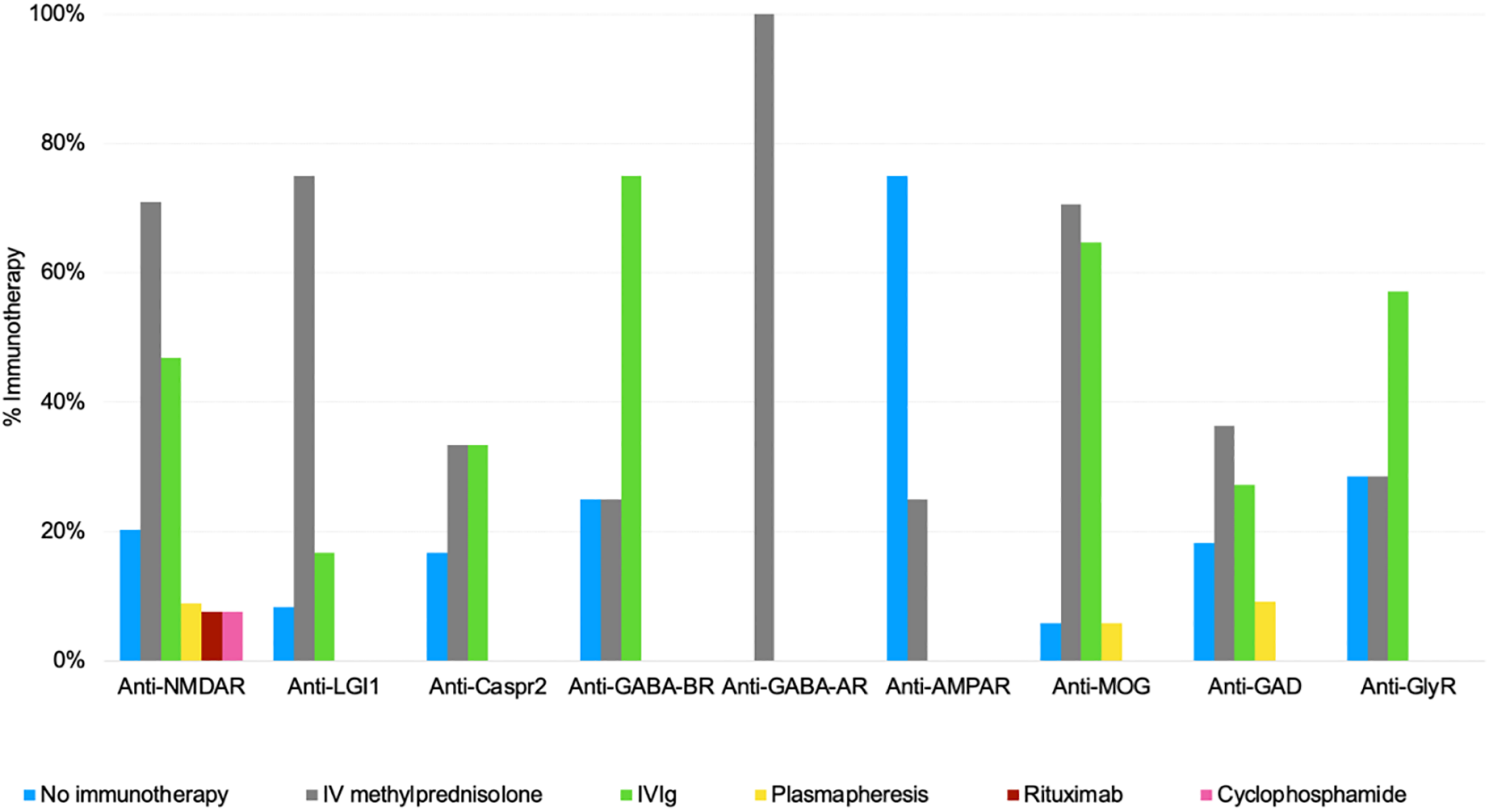
The treatment protocol used by each AIE phenotype. NMDAR, N-methyl-D-Aspartate receptor; LGI1, Leucine-rich glioma inactivated 1; CASPR2, Contactin-associated protein 2; GABA-BR, Gamma-aminobutyric acid-B receptor; GABA-AR, Gamma-aminobutyric acid-A receptor; AMPAR, a-amino-3-hydroxy-5-methyl-4-isoxazole propionic acid receptor; MOG, Myelin oligodendrocyte glycoprotein; IgLON5, Immunoglobulin-like cell adhesion molecule 5; GAD, Glutamic acid decarboxylase; GlyR, Glycine receptor; IVIg, Intravenous immunoglobulin; IV, intravenous.

All SP patients were screened for tumors at diagnosis; only 6% had neoplasia (n=9). The most common tumors were thymomas (33%), and ovarian teratomas (22%). Four patients received oncological treatment (tumor resection, chemotherapy, and/or radiation therapy). Notably, in 4 patients neurological symptoms occurred before tumor diagnosis. Among the 79 anti-NMDA encephalitis patients, only one had ovarian teratoma, which was a striking finding. **Figure 3** illustrates the treatment prescribed for each SP AIE phenotype. We observed that SP patients rarely received second-line therapy such as rituximab and cyclophosphamide, especially in anti-NMDAR cases.

Among SN patients with available treatment data (56/69), 38 patients received methylprednisolone, 26 IVIg, 5 plasmapheresis, 5 rituximab, 4 cyclophosphamide, and 14 received no treatment at enrollment.

Although in this study we did not systematically evaluate the final diagnosis of non-AIE patients, some patient information was available. Alternative diagnoses reported were epilepsy (n=8), rapidly progressive dementia (n=5), Creutzfeldt-Jakob disease (n=5), psychiatric disorder (n=4), systemic lupus erythematosus (n=3), immune-mediated ataxia (n=3), parkinsonism (n=2), Alzheimer’s disease (n=2), frontotemporal dementia (n=2), Behçet’s disease (n=1), normal pressure hydrocephalus (n=1), amyotrophic lateral sclerosis (n=1), HaNDL syndrome (Headache with neurological deficits and CSF lymphocytosis) (n=1), thyrotoxic crisis (n=1), ADEM (n=1), neuromyelitis optica spectrum disorder (n=1), multiple sclerosis (n=1), and unknown (n=20).

### Comparison of seropositive and seronegative AIE patients

Patients with SN AIE have inflammation detected in brain MRI (66/67, 98%), CSF cells (54/63, 86%), and OBC (4/14, 28%). Detailed information on SN AIE is available in **Table 3**.

**Table 3:** Comparison between seropositive and seronegative AIE patients in a Brazilian cohort.

Pediatric patients (n)	AIE		p-value
	Seronegative (15)	Seropositive (42)	
*Age (mean ± SD)*	7.27 *±* 3.32	6.26 ± 2.7	0.24
Clinical manifestations n (%)			
BPC	7 (47)	34 (81)	0.01*
Memory disturbances	1 (7)	16 (38)	0.02*
Seizures	7 (47)	31 (74)	0.06
Movement disorders	4 (27)	30 (71)	0.003*
Dystonia	1 (7)	17 (40)	0.02*
Dyskinesia	1 (7)	14 (33)	0.05*
Chorea	0 (0)	11 (26)	0.03*
Language disturbances	4 (27)	29 (69)	0.005*
DLC	7 (47)	34 (81)	0.01*
MRI findings n (%)			
MTH	3 (20)	5 (12)	0.45
WMH	7 (47)	6 (14)	0.009*
CH	4 (27)	13 (31)	0.77
BH	2 (13)	4 (9)	0.66
EEG findings n (%)			
FD	3 (20)	11 (26)	0.64
ES	3 (20)	2 (5)	0.08
EDB	0 (0)	5 (12)	0.16
GD	2 (13)	0 (0)	0.36
Pleocytosis (%)			
CSF cell account (mean ± SD)	98.1 ± 219.24	20.35 ± 38.8	0.03*
Abnormal CSF n (%)	12/13 (92)	22/39 (56)	0.01*

Adult patients (n)	AIE		p-value
	Seronegative (54)	Seropositive (103)	
Age (mean ± SD)	45.17 ± 18.12	34.33 ± 20.33	0.001*
Clinical manifestations n (%)			
BPC	45 (83)	97 (94)	0.02*
Memory disturbances	28 (52)	69 (67)	0.07
Seizures	24 (44)	79 (77)	<0.0001*
Movement disorders	13 (24)	69 (67)	<0.0001*
Orofacial dyskinesia	3 (5)	26 (25)	0.002*
Dystonia	2 (4)	22 (21)	0.005*
Dyskinesia	2 (4)	18 (17)	0.02*
Abnormal posture	2 (4)	16 (15)	0.04*
Chorea	1 (2)	8 (8)	0.13
Language disturbances	21 (39)	46 (45)	0.5
DLC	28 (52)	48 (46)	0.5
Brain MRI findings n (%)			
MTH	25 (46)	26 (25)	0.007*
WMH	15 (28)	12 (13)	0.02
CH	19 (35)	14 (10)	0.0001*
BH	11 (20)	0 (0)	0.0006*
EEG findings n (%)			
FD	10 (18)	22 (18)	1
ES	4 (7)	5 (6)	0.8
EDB	0 (0)	10 (9)	0.02*
GD	1 (2)	2 (2)	1
CSF n (%)			
CSF cell account (mean ± SD)	43.24 ± 89.14	19.26 ± 31.45	0.01*
Abnormal CSF n (%)	42/50 (84)	42/101 (41)	<0.0001*

*p < 0.05.

BPC, Behavioral and psychiatric changes; DLC, Decreased level of consciousness; MTH, Mesial temporal hyperintensities; CH, Cortical hyperintensities; WMH, White matter hyperintensities; BH, Brainstem hyperintensities; FD, Focal discharges; ES, Electrographic seizures; EDB, Extreme Delta-Brush; GD, Generalized discharges.

The comparison of pediatric SN and SP AIE showed that SP pediatric patients had more frequent behavioral and psychiatric changes, memory disturbances, movement disorders (such as chorea, dystonia, dyskinesia), language disturbances, and decreased level of consciousness. SN AIE patients presented more frequent white matter hyperintensities and higher pleocytosis than SP AIE children. We did not find differences between groups in other clinical variables and EEG findings (**Table 1**).

The comparison of adult SN and SP AIE showed that SP patients were younger, and had more behavioral and psychiatric changes, seizures, movement disorders (such as orofacial dyskinesia, dystonia, dyskinesia), and abnormal posture. Brain MRI abnormalities were more frequent in SN patients. CSF WBCs were lower in ​​SP adults, and we found a higher frequency of extreme delta-brush among SP adult AIE patients. We did not find any differences between groups in other clinical variables (**Table 1**).

### Predictors of AIE among adult and pediatric patients

Clinical variables associated with SP AIE in the pediatric population were decreased level of consciousness and chorea (**Table 2**). In the adult population, variables associated with SP AIE were movement disorders, seizures, autonomic instability, and memory impairment (**Table 4**).

**Table 4:** Predictors of AIE in pediatric and adult patients.

Pediatric patients	p-value	Exp(B)	95% CI	
*BPC*	0.875	0.896	0.229	3.513
*DLC*	0.040*	3.005	1.051	8.595
*Memory disturbances*	0.879	1.127	0.243	5.219
*Language disturbances*	0.167	1.930	0.759	4.907
*Movement disorders*	0.288	1.692	0.641	4.464
Chorea	0.002*	8.985	2.227	36.246
Dyskinesia	0.654	1.306	0.407	4.191
Orofacial dyskinesia	0.952	0.958	0.235	3.897
Adult patients	p-value	Exp(B)	95% CI	
*BPC*	0.997	1.002	0.341	2.947
*Memory disturbances*	0.001*	2.747	1.543	4.890
*Seizures*	0.000*	4.524	2.537	8.067
*Language disturbances*	0.579	0.849	0.477	1.511
*Autonomic instability*	0.026*	2.128	1.096	4.131
*Movement disorders*	0.000*	4.130	2.359	7.228
Chorea	0.850	0.889	0.261	3.024
Dyskinesia	0.700	1.230	0.430	3.512
Orofacial dyskinesia	0.643	0.800	0.312	2.054

BPC, Behavioral and psychiatric changes; DLC, Decreased level of consciousness. * p-value < 0.05.

## Discussion

In Brazil, the most common antibodies associated with AIE among adults are anti-NMDAR, followed by anti-LGI1, anti-GAD, anti-GlyR, anti-Caspr-2, and anti-AMPAR. These findings align with a large report of 41,217 adults in the United States that reported anti-NMDAR, anti-GAD65, and anti-LGI1 as the most frequent antibodies in serum and CSF (12). However, our results differ from small reports in Hungary (22), China (10, 23), and Iran (11). This might be attributed to the smaller samples in those studies, techniques used for ANeA detection, and potential genetic susceptibilities in different populations, such as the Brazilian admixed population. Some AIE subtypes have specific HLA susceptibility genes (24–26) that have been reported in some populations but not in others, findings which were also reported in neuromyelitis optica and multiple sclerosis (27, 28).

Our results from pediatric patients are in line with prior data from the Spanish, Danish, and Chinese populations, who identified anti-NMDAR and anti-MOG as the most common antibodies among children with possible AIE (29–33). We found only one child with anti-GlyR antibodies that presented with psychosis, abnormal posture, and ataxia. Other antibodies such as anti-AMPAR, anti-LGI1, anti-Caspr2, and anti-GABA-AR were rarely reported in the pediatric population (29–36). Therefore, our findings together with the prior data might indicate that cost-effective panels prioritizing anti-NMDAR and anti-MOG antibodies in children might be a reasonable approach to diagnosis, especially in low-income countries, and to early refer samples to research labs if the child has negative anti-MOG and anti-NMDAR results and fulfill the clinical criteria for pediatric AIE.

In this study, we found anti-GlyR antibodies in 7% of the SP AIE cases without co-occurrence with other antibodies. Anti-GlyR antibodies were initially described in progressive encephalomyelitis with rigidity and myoclonus (PERM) (37). Nonetheless, since 2018 anti-GlyR has been also reported in the serum and/or CSF of patients with epilepsy, cerebellar ataxia, parkinsonism, optic neuritis, multiple sclerosis, ADEM, and Bickerstaff, which may indicate that the antibody is unspecific and not associated with a detailed clinical phenotype (38–42). Additionally, a cohort from Australia reported anti-GlyR in over 20% of patients with possible AIE (38, 43). In our study, all anti-GlyR patients were carefully selected using the AIE criteria, all developed abnormal movements and were screened for other antibodies, including moderate and high-risk antibodies in CSF and serum. Although anti-GlyR antibodies were positive only in serum, and one should interpret these results with caution, such finding has been described in other series (38, 43). We believe our results may help foster the discussion on the relevance of anti-GlyR in AIE and how to interpret positive results. Despite anti-GlyR being also described in other diseases, we believe that in the context of AIE with brainstem involvement and movement disorder, its detection might indicate an immune-mediated pathophysiology. Whether the antibody is truly pathogenic remains unclear. Because anti-GlyR antibodies are not detected in TBA, commercial kits might underestimate their prevalence. Future studies should evaluate whether systematic testing for anti-GlyR is a reasonable strategy when investigating patients with possible AIE.

Another important result from this study is the incidence of anti-MOG in adult patients with AIE. We found that 3% of the adult patients with AIE harbor anti-MOG antibodies which is higher than other antibodies such as anti-GABA-AR and anti-GABA-BR. Anti-MOG encephalitis in adults typically presents with cortical encephalitis, characterized by fever, headache, seizures, and brain MRI FLAIR-hyperintensities (FLAMES) (44, 45), while others report a clinical presentation very similar to ADEM, with a decreased level of consciousness and movement disorders (46–49). We report three cases, with clinical presentation not suggestive of demyelinating symptoms, including a later presentation. Further studies should investigate and confirm the occurrence of anti-MOG encephalitis in adults, especially because treatment and prognosis of anti-MOG may differ from AIE.

Our study confirms that in some populations the proportion of males with anti-NMDAR may reach 30% and association with teratoma and malignancy may vary. Anti-NMDAR encephalitis was mainly seen in young females with a high percentage of cases with an ovarian teratoma (50–53) around 50%, especially in the Caucasian population. We found similar results to the Australian and Chinese cohorts that reported 25% of males and only 10% association with tumors (10, 43). Future genetic analysis of a multiethnic population will clarify this pending question.

We provide consistent information from ANPRA, as all cases classified as seronegative were reviewed and tested with TBA and CBA in both serum and CSF, avoiding misdiagnosis (21). We observed that of the 564 patients with possible AIE, 12% met the criteria for ANPRA phenotype. This specific group of patients was rarely submitted to brain biopsy. We observed that pediatric SN AIE patients were more frequently male and had fewer movement disorders than SP AIE patients (54). Among adults, we found that SN patients were older, and had less cognitive involvement and abnormal movements. Because of those variable clinical manifestations future prospective studies should evaluate the final diagnosis of these patients. We also provide evidence that patients who fulfill the possible AIE criteria may have another disease such as CJD, SLE and other inflammatory diseases, AD, and other degenerative diseases, highlighting the importance of adequate testing to exclude AIE and further look for alternate diagnosis.

Seasonal variation in the onset of autoimmune diseases may reflect changes in external environmental factors, some of which hold the potential to modulate pathogenic processes involved in autoimmunity (55). Prior transversal studies from centers of non-tropical countries supported a higher frequency of AIE in warmer months (16, 17). Although we found more cases in summer, statistical analysis for seasonality was not significant after approximately four years of follow-up.

We report a delay in AIE diagnosis in Brazil by several months, which is longer than other cohorts with a median delay of around 1 month (4). Mainly, this reality is due to limited access to testing, the extensive investigation to rule out a differential diagnosis that mimics AIE (18, 56–58), and the lack of knowledge about the disease among clinical and neurology staff. Although we did not perform a prospective study on outcomes, treatment options, and final diagnoses of the whole cohort, our results might indicate that we prescribe less frequently second-line therapy in confirmed AIE, indicating a barrier to treatment with high-cost medications. Since the appropriate use of resources is critical in low-income countries, we suggest not to test patients when possible AIE criteria are not met. Considering that the estimated prevalence of AIE is higher than neuromyelitis optica spectrum disorder (1-10/100.000) (59), future studies should address cost-effective autoantibody testing for AIE.

The strengths of this study are multicentric design and comprehensive testing with TBA and CBA including systematic screening for anti-GlyR and anti-MOG antibodies. The use of additional CBAs for anti-MOG and anti-GlyR, which are antibodies not detected in TBAs, yielded 6,9% of the cases in these series. However, limitations of the study include its cross-section design, lack of follow-up data, and cost-effectiveness analysis. We identified certain clinical variables that may aid in predicting AIE; in children, the presence of decreased level of consciousness, chorea, and dystonia is associated with AIE. Among adults, memory impairment and movement disorders indicate AIE. We believe these findings can assist clinicians in navigating the clinical scenario and making decisions regarding antibody testing and treatment. However, one exception would be when anti-IgLON5-associated encephalitis is suspected, as the clinical course could be protracted.

In summary, our study provides comprehensive clinical data on both adult and pediatric AIE patients and challenges from developing countries. Our results suggest that anti-GlyR and anti-MOG antibodies may be considered alongside other surface and synaptic antibodies in the cases of AIE. Clinical variables such as memory complaints, decreased level of consciousness, and movement disorders may help navigate the clinical decisions and treatment in patients with possible AIE.

## Data availability statement

The raw data supporting the conclusions of this article will be made available by the authors, without undue reservation.

## Ethics statement

The studies involving humans were approved by Medical Research Council of Hospital Israelita Albert Einstein. The studies were conducted in accordance with the local legislation and institutional requirements. Written informed consent for participation in this study was provided by the participants’ legal guardians/next of kin.

## Author contributions

BF: Investigation, Conceptualization, Data curation, Formal Analysis, Methodology, Project administration, Writing – original draft, Writing – review & editing. FF: Investigation, Conceptualization, Data curation, Formal Analysis, Methodology, Project administration, Writing – original draft, Writing – review & editing. MES: Methodology, Writing – review & editing. RA: Methodology, Writing – review & editing. AD: Methodology, Resources, Writing – review & editing. PK: Methodology, Writing – review & editing. HT: Methodology, Writing – review & editing. MS: Methodology, Writing – review & editing. AF: Methodology, Writing – review & editing. LJ: Methodology, Writing – review & editing. PB-N: Methodology, Writing – review & editing. PR: Methodology, Writing – review & editing. JO-F: Methodology, Writing – review & editing. RM: Methodology, Writing – review & editing. CO: Methodology, Writing – review & editing. FM: Methodology, Writing – review & editing. RB: Methodology, Writing – review & editing. MLS: Writing – review & editing, Methodology. ES: Methodology, Writing – review & editing. AN: Methodology, Writing – review & editing. KL: Methodology, Writing – review & editing. OG: Methodology, Writing – review & editing. VE: Writing – review & editing, Methodology. LC: Conceptualization, Funding acquisition, Methodology, Resources, Writing – review & editing. RH: Resources, Conceptualization, Funding acquisition, Investigation, Methodology, Writing – original draft, Writing – review & editing. LA: Conceptualization, Funding acquisition, Investigation, Methodology, Project administration, Supervision, Validation, Visualization, Writing – original draft, Writing – review & editing.

## Supplementary material

Supplementary Figure Antibodies profile of Brazilian patients with AIE. NMDAR, N-methyl-D-Aspartate receptor; LGI1, Leucine-rich glioma inactivated 1; CASPR2, Contactin-associated protein 2; GABA-BR, Gamma-aminobutyric acid-B receptor; GABA-AR, Gamma-aminobutyric acid-A receptor; AMPAR, a-amino-3-hydroxy-5-methyl-4-isoxazole propionic acid receptor; MOG, Myelin oligodendrocyte glycoprotein; IgLON5, Immunoglobulin-like cell adhesion molecule 5; GAD, Glutamic acid decarboxylase; GlyR, Glycine receptor.
