# Supplementary figures and images for: Brazilian autoimmune encephalitis network (BrAIN): antibody profile and clinical characteristics from a multicenter study
Source: Front Immunol. 2023 Oct 25;14:1256480. doi: 10.3389/fimmu.2023.1256480 (PMC10634608; doi:10.3389/fimmu.2023.1256480)

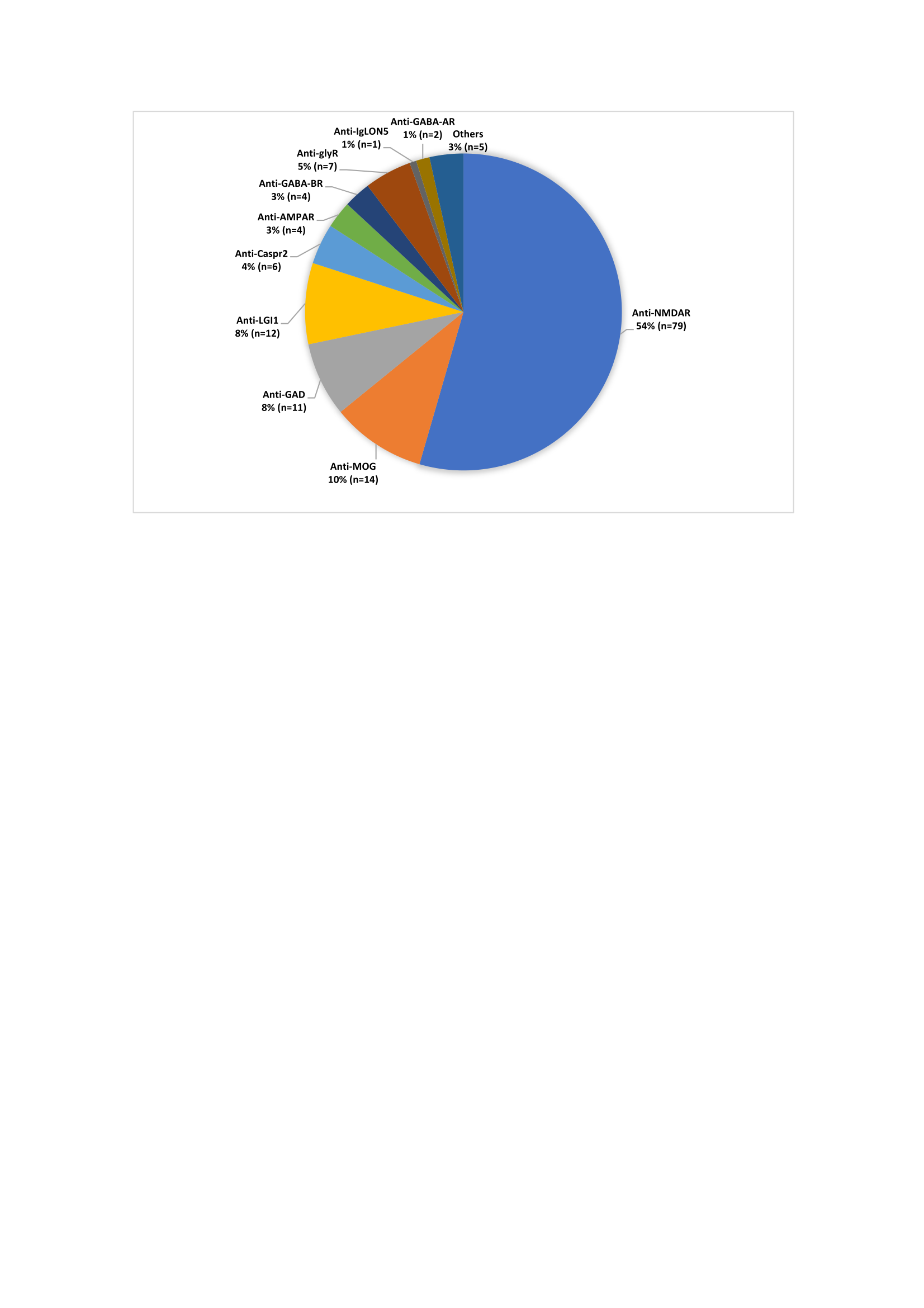

Supplement: Supplementary Figure — Antibodies profile of Brazilian patients with AIE. NMDAR, N-methyl-D-Aspartate receptor; LGI1, Leucine-rich glioma inactivated 1; CASPR2, Contactin-associated protein 2; GABA-BR, Gamma-aminobutyric acid-B receptor; GABA-AR, Gamma-aminobutyric acid-A receptor; AMPAR, a-amino-3-hydroxy-5-methyl-4-isoxazole propionic acid receptor; MOG, Myelin oligodendrocyte glycoprotein; IgLON5, Immunoglobulin-like cell adhesion molecule 5; GAD, Glutamic acid decarboxylase; GlyR, Glycine receptor. [file Image_1.tiff]
